# *In vitro* and *in vivo* immunomodulatory properties of octyl-β-d-galactofuranoside during *Leishmania donovani* infection

**DOI:** 10.1186/s13071-019-3858-0

**Published:** 2019-12-23

**Authors:** Hélène Guegan, Kevin Ory, Sorya Belaz, Aurélien Jan, Sarah Dion, Laurent Legentil, Christelle Manuel, Loïc Lemiègre, Thomas Vives, Vincent Ferrières, Jean-Pierre Gangneux, Florence Robert-Gangneux

**Affiliations:** 1 CHU Rennes, Inserm, EHESP IRSET (Institut de Recherche en Santé Environnement et Travail) – UMR_S 1085, University of Rennes, 35000 Rennes, France; 2 Inserm, EHESP, IRSET (Institut de Recherche en Santé Environnement et Travail) – UMR_S 1085, University of Rennes, 35000 Rennes, France; 3 Ecole Nationale Supérieure de Chimie, CNRS, UMR 6226, University of Rennes, avenue du Général Leclerc CS 50837, 35708 Rennes cedex 7, France

**Keywords:** Visceral leishmaniasis, *Leishmania donovani*, Immunostimulation treatment, Galactofuranose, Furanoside, Macrophage polarization

## Abstract

**Background:**

The chemotherapeutic arsenal available to treat visceral leishmaniasis is currently limited, in view of many drawbacks such as high cost, toxicity or emerging resistance. New therapeutic strategies are particularly needed to improve the management and the outcome in immunosuppressed patients. The combination of an immunomodulatory drug to a conventional anti-*Leishmania* treatment is an emerging concept to reverse the immune bias from Th2 to Th1 response to boost healing and prevent relapses.

**Methods:**

Here, immunostimulating and leishmanicidal properties of octyl-β-d-galactofuranose (Galf) were assessed in human monocyte-derived macrophages (HM) and in a murine model, after challenge with *Leishmania donovani* promastigotes. We recorded parasite loads and expression of various cytokines and immune effectors in HM and mouse organs (liver, spleen, bone marrow), following treatment with free (Galf) and liposomal (L-Galf) formulations.

**Results:**

Both treatments significantly reduced parasite proliferation in HM, as well as liver parasite burden *in vivo* (*Galf, P* < 0.05). Consistent with *in vitro* results, we showed that Galf- and L-Galf-treated mice displayed an enhanced Th1 immune response, particularly in the spleen where pro-inflammatory cytokines TNF-α, IL-1β and IL-12 were significantly overexpressed compared to control group. The hepatic recruitment of myeloid cells was also favored by L-Galf treatment as evidenced by the five-fold increase of myeloperoxidase (MPO) induction, which was associated with a higher number of MPO-positive cells within granulomas. By contrast, the systemic level of various cytokines such as IL-1β, IL-6, IL-17A or IL-27 was drastically reduced at the end of treatment.

**Conclusions:**

Overall, these results suggest that Galf could be tested as an adjuvant in combination with current anti-parasitic drugs, to restore an efficient immune response against infection in a model of immunosuppressed mice.
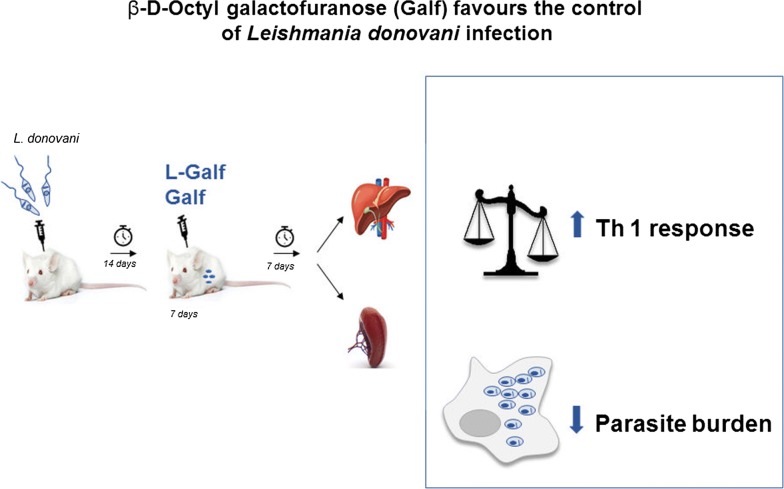

## Background

Leishmaniasis is a neglected disease endemic in 98 countries, ranked by the World Health Organization as the second most important human protozoan parasitic disease after malaria [1]. The estimated incidence of visceral leishmaniasis (VL), the most severe clinical form of the disease, is 0.5 million human cases. Besides, canine symptomatic and asymptomatic VL contributes to the spread of *Leishmania* [2] and represents a public health concern, as the seroprevalence in dogs is estimated to be about 40% in endemic areas [3–5]. After inoculation by the sand fly vector, parasites infect macrophage cells and other phagocytic cells (neutrophils, dendritic cells) and diffuse to lymphoid organs, with the spleen, the bone marrow, the liver, and lymph nodes being the targeted tissues. *Leishmania* parasites replicate inside macrophage cells and can downmodulate the host immune response to persist until host death, if left untreated [6]. Conventional therapies used for the treatment of VL require prolonged administration, and/or have toxicity risks or are currently facing challenges due to drug resistance in endemic regions [7]. New therapeutic agents, i.e. liposomal amphotericin B and miltefosine, have demonstrated their efficacy in large field clinical trials. However, their widespread use is limited by adverse events, cost and intravenous use, and thus stress the need to find new targets or to investigate novel cost-effective therapeutic approaches. Additionally, in India, the development of complications such as post-kala-azar dermal leishmaniasis (PKDL) is a major issue and still poorly understood, but probably involves improper immune response [8, 9]. The strategy of immunostimulation combined to anti-parasitic treatment is an attractive approach to circumvent treatment failures, particularly in immunocompromised hosts, who experience frequent relapses [10].

The proof of concept of immunomodulation has been investigated in several *in vitro* studies [11–13], and in human cohorts, mainly by blocking the IL-10 pathway [14–16], by IFN-γ or IL-2 supplementation [17, 18], or by various antigens [10, 19]. Indeed, *Leishmania* parasites can interfere with cell signaling to downmodulate the host immune response, and to persist within cells and replicate until host death, if left untreated. Among several lines of explanations [20], it has been proposed that *Leishmania* could favor the differentiation of macrophages into a M2 phenotype, which is permissive to parasite persistence [21, 22], thus could be targeted for stimulation and reprogramming towards M1 phenotype.

The *Leishmania* cell membrane is mainly composed of lipophosphoglycans (LPG) and glycosylinositol phospholipids (GIPLs), which contribute to parasite virulence, cell invasion and interference with host cell signaling [23], with possible differences according to GIPLs structure [24]. The observation of an increase in cured patients after infection with killed promastigotes associated to conventional treatment [25] suggested that immunization or therapeutic vaccination could also contribute to strengthen treatment efficacy in cutaneous leishmaniasis, and possibly in VL [26].

LPG and GIPLs have the peculiarity to contain furanose motifs, which are absent from the mammalian cell membrane [27]. For several years, our consortium has investigated the innovative chemical synthesis of galactofuranose derivatives, and we have previously shown that octyl β-D-galactofuranoside (Galf) has significant *in vitro* anti-leishmanial activity, comparable to the reference compound miltefosine, leading to important structure damage in promastigotes [28]. Furthermore, we provided evidence that Galf is able to induce reactive oxygen species (ROS) production in human macrophages *in vitro*, limiting parasite proliferation [28]. According to the literature, galactopyranosides and/or LPG can interact with various receptors (mannose receptor, SIGN-R1, DC-SIGN, and toll-like receptor 2 present on macrophages, which could lead to immunostimulation [22, 29]. Since the mode of action of Galf on infected macrophages remained to be elucidated, we investigated its potential effect as an immunomodulator.

Therefore, the aim of this study was to evaluate the potential anti-*Leishmania* and immunomodulatory properties of Galf *in vitro* and *in vivo*, in the context of *L. donovani* infection. Additionally, we evaluated a liposomal formulation of Galf in the aim to better target macrophage cells *in vivo.*

## Methods

### Mice

Female BALB/c wild-type mice, aged 7 weeks (18–25g) were purchased from Janvier Laboratories (Le Genest-Saint-Isle, France) and housed in level A3 confinement in our animal facility (agreement number #B35-238-40). A 7-day acclimatization period before challenge was respected.

### *Leishmania* culture and maintenance

The *L. donovani* strain used in this study was kindly provided by Dr A. Descoteaux (Institut National de la Recherche Scientifique, Quebec, Canada) and was originally isolated from a Sudanese visceral leishmaniasis patient (LV9 Sudanese strain 1S). Prior to infection, amplification of promastigotes was carried out by culture in modified M199 medium (Sigma-Aldrich, Saint Quentin Fallavier, France) supplemented with 10% inactivated fetal calf serum, 10 mM HEPES, 100 µM hypoxanthine, 5 µM hemin, 1 µM biotin, 4 µM biopterin, 100 IU/ml penicillin and 100 µg/ml streptomycin (Gibco, Waltham, MA, USA) pH 7.4 at 27 °C for 6 days, until they reached stationary phase.

### Galactofuranoside formulations

Octyl β-D-galactofuranoside (Galf) used for *in vitro* and *in vivo* experiments was synthesized as already described [30], filtered and lyophylized. Prior to experiments, it was reconstituted to 5 mg/ml (Galf treatment) with 1× DPBS (Gibco Life Technologies). The Galf liposome formulations were prepared with the following composition: EggPC/Chol/DSPE-PEG_2000_: 75:20:5. Lipid mixtures were prepared at a final concentration of 5 mg/ml by dissolving the required amount of lipid and octyl galactofuranoside (2 mg/ml) in chloroform. The organic solvents were removed under reduced pressure to form a lipid film, which was further dried overnight under vacuum to remove traces of the solvents. Liposomes were formed by hydration of the lipid films with 1× DPBS at RT and were stored at 4 °C for 24 h. Formulations were sonicated at 40 °C for 5 min using an ultrasonic bath at 37 kHz. The encapsulation efficiency was determined by LCMS analysis after centrifugation filtration of the liposome formulation. The liposomal concentrate contained more than 70% of Galf molecule initially introduced. Liposome formulations (L-Galf treatment) were stable for more than 15 days at 4 °C. Liposomal amphotericin B (Gilead Sciences, Boulogne-Billancourt, France), meglumine antimoniate (Sanofi Aventis, Gentilly, France), and miltefosine (Baxter SAS, Maurepas, France) (HePC) were used as reference treatments.

### Human macrophage cultures

Human blood monocytes-derived macrophages (HM) were obtained by purifying monocytes from peripheral blood mononuclear cells obtained from blood buffy coats (supplied by Etablissement Français du Sang, Rennes, France), as described earlier [31]. Briefly, cells were cultured in RPMI 1640 medium (Gibco Life Technologies) supplemented with 10% decomplemented fetal calf serum (FCS), 100 IU/ml penicillin and 100 µg/ml streptomycin and differentiated with M-CSF (100 ng/ml) for 6 days to obtain primary human M0 macrophages. Cells were seeded in 8-well plates (Labtech, Palaiseau, France) for parasite growth quantification and in 6-well plates for mRNA quantification and incubated at 37 °C with 5% CO_2_.

Macrophages were infected overnight with *L. donovani* promastigotes (MOI 10:1) at stationary phase. After a washing step with RPMI, cells were treated with Galf at 80 µM, or with sterile PBS (untreated DPBS control group). For mRNA quantification of cytokines and immune markers, cells were washed after 24 h of treatment and lysed immediately before extraction using QiampRNA mini-kit (Qiagen, Courtaboeuf, France). Subsequent reverse transcription and quantification of mRNA induction of cytokines and immune markers were performed as described below for mouse liver and spleen. For the parasite growth assay, cells were treated with Galf at various concentrations (5 µM, 10 µM, 20 µM, 40 µM or 80 µM) or L-Galf (10 µM, 20 µM, 40 µM or 80 µM), or reference molecules (miltefosine (HePC) at 8 µM and meglumine antimoniate (Gluc) at 100 µg/ml) for 48 h, or left untreated. After incubation, cells were washed, dried and stained with May-Grünwald-Giemsa (MGG), and observed by optical microscopy at a 100× magnification. Quantification of parasite burdens was evaluated by counting the percent of infected cells and the number of intracellular parasites in treated and untreated control cells. Each condition was performed in quadruplicate and each experiment was repeated 2 to 4 times, depending on the concentrations.

### Protocol of mouse experiments

BALB/c mice were infected intraperitoneally with 1.10^8^ *L. donovani* promastigotes grown for 6 days in culture with 500 µl of DPBS, as previously described [32]. Fourteen days after infection (day 14), animals were treated daily by intraperitoneal route, for 7 days. Mice were separated into 5 treatment groups (*n* = 9 or 10 mice per group), consisting of: (i) 1× DPBS (control group) daily; (ii) 100 µg (i.e. 5 mg/kg body weight) of liposomal amphotericin B (L-AmB) every other day; (iii) 93 µg (i.e. 4.6 mg/kg) of free Galf daily; (iv) 93 µg (i.e. 4.6 mg/kg) of liposomal Galf (L-Galf) included in 800 µg of liposome daily; or (v) 800 µg (i.e. 40 mg/kg) of empty liposomes (Lipo) daily. All treatments were given in a volume of 400 µl. Blood samples were collected from the sub-mandibulary vein at end of treatment (day 21), immediately centrifuged and serum was frozen at −80 °C. Animals were sacrificed 7 days after the end of treatment (day 28).

### RNA isolation and analysis of gene expression in organs

Total cellular RNA was extracted and purified from liver and spleen samples using TRI Reagent^TM^ Solution (Invitrogen, Paris, France) and then treated with DNase (Promega, Charbonnières-les-Bains, France) (1 U DNase/µg total RNA). RNA from bone marrow cells (BM) was extracted using the Nucleospin® RNA kit (Macherey-Nagel, Hoerdt, France). Reverse transcription was performed with a high-capacity cDNA reverse transcription kit (Applied Biosystems, Villebon-sur-Yvette, France) according to the manufacturer’s instructions.

Quantitative PCR amplifications were carried out in duplicate using Power SYBR^®^ green PCR master mix (Applied Biosystems), 0.3 µM primers, and 2 µl of cDNA in a final volume of 10 µl, in 384-well optical plates, using a CFX384 Touch^TM^ real-time PCR detection system (Bio-Rad, Marnes-la-Coquette, France). Gene-specific primers (Additional file 1: Table S1) were synthesized by Sigma-Aldrich (Lyon, France). Expression levels of target genes were normalized by comparison to expression of murine *18S* rRNA. Results were expressed as 2^–ΔΔCq^ referring to the fold induction in relation to the mean quantification cycle obtained with DPBS-treated mice.

### Quantification of parasite loads in liver and spleen

Parasite burdens were determined by a limiting dilution technique adapted from Buffet et al. [33]. For this, a piece of spleen and liver was excised, weighed and homogenized with 2 or 4 ml of 1× Schneider’s Drosophila medium (Gibco), supplemented with 20% inactivated FCS, 100 µM hypoxanthine, 100 IU/ml penicillin and 100 µg/ml streptomycin (Gibco), respectively, using a tissue grinder. Under sterile conditions, each sample was plated in duplicate and serial 4-fold dilutions ranging from 1 to (1:4)^11^ were prepared in 96-well microtitration plates containing 225 µl of culture medium. Microscopic examination of cultures was performed at day 7 and day 14 after incubation at 27 °C, and results were expressed as log (titer: organ weight (mg)), with the titer corresponding to the dilution of the last positive well.

### Immunohistochemistry

#### Myeloperoxidase staining in liver sections

Immunohistochemistry was performed on the Discovery Automated IHC stainer using the Ventana DABMap detection kit (Ventana Medical Systems, Tucson, Ariz). Following deparaffinization with Ventana EZ Prep solution at 75 °C for 8 min, antigen retrieval was performed using Tris-based buffer solution CC1 (Ventana Medical Systems) at 95 °C to 100 °C for 12 min. Endogen peroxidase was blocked with Inhibitor-D 3% H2O2 (Ventana) for 8 min at 37 °C. After rinsing with reaction buffer (Roche, Meylan, France), slides were incubated at 37 °C for 32 min with an appropriate dilution of primary anti-myeloperoxidase antibodies. After rinsing, signal enhancement was performed using the Ventana DABMap Kit and secondary antibody: biotinylated horse anti-rabbit (Vector laboratory, Burlingame, CA, USA) was incubated for 32 min. Slides were then counterstained for 16 min with hematoxylin, 4 min with bluing reagent, and rinsed. After removal from the instrument, slides were manually dehydrated and a glass coverslip applied.

#### Immunohistochemical characterization of immune cells in the spleen

Paraffin-embedded tissue was cut at 4 µm, mounted on positively charged slides and dried at 58 °C for 60 min, then processed as described above and incubated with primary antibodies (rat anti-mouse CD8, rat anti-mouse CD4, and rat anti-mouse CD20 all from Santa Cruz Biotechnology, Inc., Heidelberg, Germany), diluted at 1:100. After rinsing, slides were incubated with appropriate donkey anti-rat secondary antibodies (Jackson ImmunoResearch, Ely, UK).

### Quantification of liver granulomas

The histological response to infection was evaluated quantitatively after microscopic examination of paraffin-embedded liver sections stained with hematoxylin and eosin (H&E). Slide images were obtained using the NanoZoomer (Hamamatsu Photonics, Massy, France), and analyzed using the NDP.view2 viewing software (Hamamatsu Photonics) for granuloma quantification and size analysis. Granulomas were classified into 2 categories based on the structure area: (i) < 3000 µm^2^ (< 50 cells); and (ii) ≥ 3000 µm^2^ (≥ 50 cells).

### Biochemical dosages

Serum alanine transaminases (ALT) and alkaline phosphatase (ALP) were measured according to the IFCC primary reference procedures, using a Cobas® analyzer (Roche), according to the manufacturer’s instructions. Serum creatinine was measured using a colorimetric method, on the same device.

### Serum cytokines dosage

Twenty-four hours after the end of treatment, blood samples were collected to quantify circulating cytokines levels. Titrations were performed using a bead-based immunoassay LEGENDplex^®^ multi-analyte flow assay kit (BioLegend, Paris, France), according to the manufacturerʼs protocol. Cytokine concentration was determined using standard curves obtained using recombinant cytokine standards provided in the kit. Samples were analyzed on a LSRFortessa^TM^ cytometer (BD Biosciences, Le-Pont-de-Claix, France).

### Statistical analysis

*In vitro* and *in vivo* data are expressed as means ± standard errors of the means (SE) for each group. Multiple comparisons between untreated group and treated groups were analyzed using the non-parametric Kruskal–Wallis test. For comparisons between 2 groups, the non-parametric Mann-Whitney test was used. Statistical analysis was performed using GraphPad Prism 6 software. Differences were considered significant when the *P*-value was ≤ 0.05 and are indicated as follows: *P ≤ 0.05, **P ≤ 0.01 and ***P ≤ 0.001.

## Results

### Galf and L-Galf treatment reduce *L. donovani* proliferation in human macrophages

**Fig. 1:**
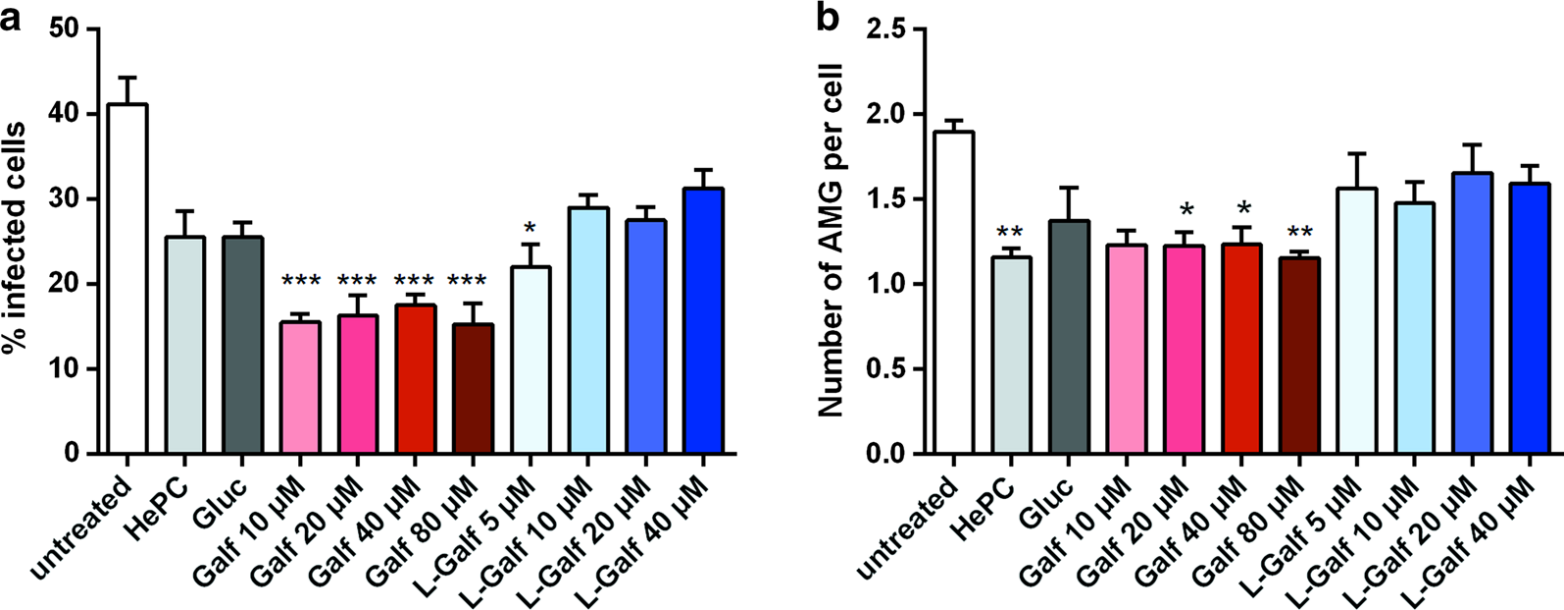
Anti-*Leishmania* effect of Galf on human macrophages *in vitro*. **a** Percentage of infected macrophages after treatment with Galf or L-Galf at various concentrations, or with 8 µM miltefosine (HePC) or 100 µg/ml meglumine antimoniate (Gluc). **b** Mean number of amastigotes (AMG) per macrophage. Human macrophages were infected overnight with *L. donovani* promastigotes (MOI 10:1) at stationary phase, then cells were treated with the indicated compounds for 48 h. After washing, cells were stained with MGG and observed by optical microscopy at a magnification of 100× to count the percentage of infected cells and the number of intracellular parasites in treated and untreated cells. Each condition was performed in quadruplicate in two independent experiments. Data show means ± SE of one representative experiment. Results from treated groups were compared to the untreated group using the non-parametric Kruskal–Wallis test (*P ≤ 0.05, **P ≤ 0.01, ***P ≤ 0.001)

The antiparasitic effect of Galf was investigated *in vitro* in human macrophages. The rate of infected cells and the number of amastigotes were determined after treatment by Galf formulations and compared to untreated condition (Fig. 1). Remarkably, the number of infected cells was reduced by half with Galf, compared to control group, whatever the concentration used (Kruskal–Wallis test: *χ*^2^ = 39.69, *df* = 10, *P* = 0.0002, *P* = 0.0003, *P* = 0.0009, *P* = 0.0002 at 10 µM, 20 µM, 40 µM and 80 µM, respectively), while it decreased with L-Galf from 41.2 ± 3.2% to 22 ± 2.7% at 5 µM (Kruskal–Wallis test: *χ*^2^ = 39.69, *df* = 10, *P* = 0.0246) (Fig. 1a). Higher concentrations yielded a similar decrease, as that obtained with reference drugs. Similarly, a reduction of the number of amastigotes per cell was observed with Galf treatments (1.23 ± 0.08 and 1.23 ± 0.100 at 20 µM and 40 µM, respectively, Kruskal–Wallis test: *χ*^2^ = 23.03, *df* = 10, *P* = 0.0335 and *χ*^2^ = 23.03, *df* = 10, *P* = 0.0319) and 1.16 ± 0.04 at 80 µM, (*χ*^2^ = 23.03, *df* = 10, *P* = 0.0064) compared to untreated control (1.90 ± 0.07) (Fig. 1b).

### Galf-treated macrophages have a mixed polarization profile

**Fig. 2:**
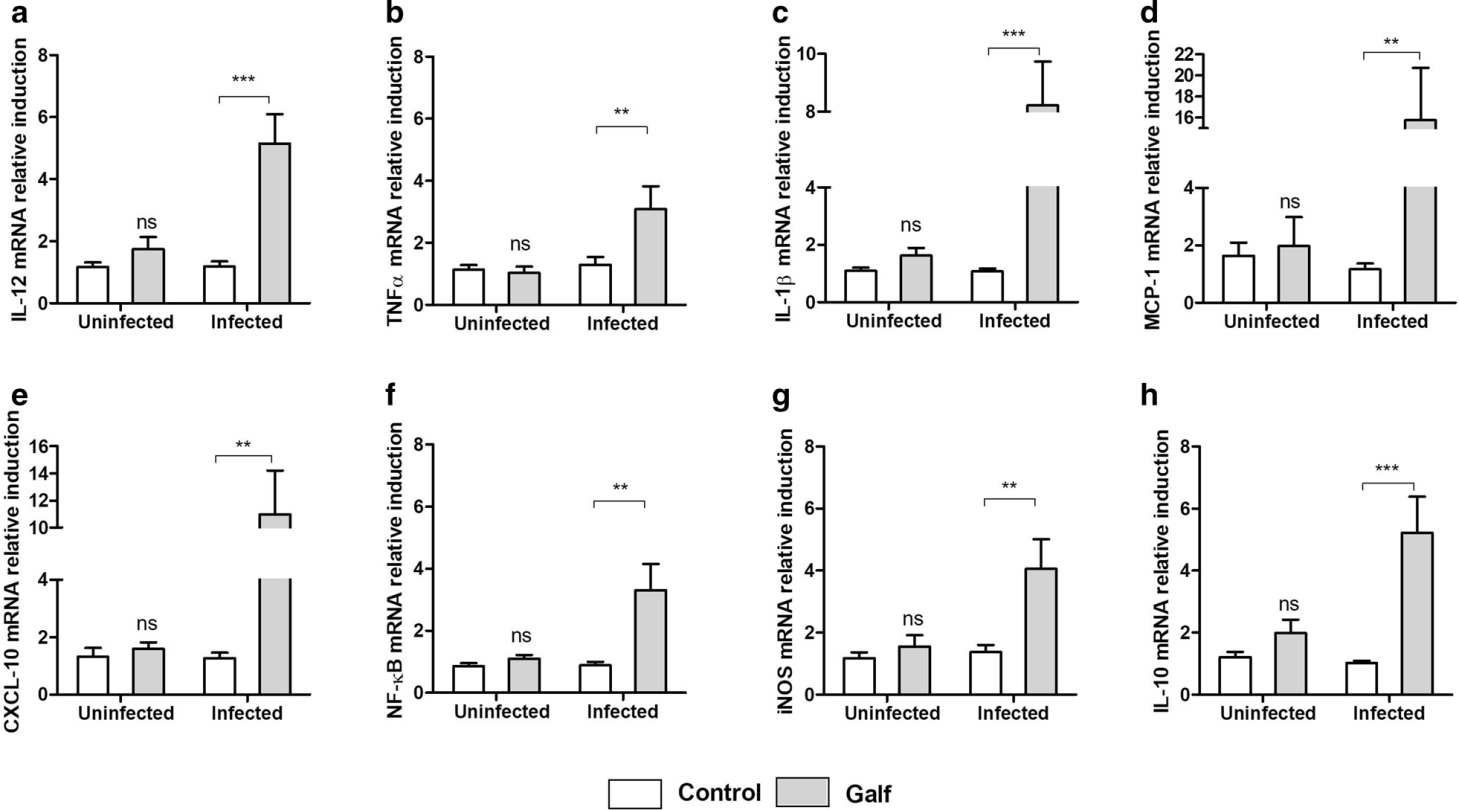
Immunomodulatory effect of Galf on human macrophages in vitro. mRNA expression of IL-12 (**a**), TNF-α (**b**), IL-1β (**c**), MCP-1 (**d**), CXCL-10 (**e**), NF-ƙB (**f**), iNOS (**g**) and IL-10 (**h**) was evaluated after 24 h of treatment by Galf. Human M0 macrophages were infected overnight with *L. donovani* promastigotes (MOI 10:1) at stationary phase, or left uninfected. Cells were treated with Galf at 80 µM for 24 h or left untreated (control group). Cells were lysed immediately before RNA extraction. After reverse transcription, mRNA induction was quantified by quantitative PCR. Data were normalized to *18S* rDNA and are representative of four independent experiments. Mean inductions ± SE are represented by cross bars. Results from treated groups were compared to the untreated group using the non-parametric Mann-Whitney test (**P ≤0.01, ***P ≤0.001)

Immunomodulatory effect of Galf was evaluated in infected and uninfected human macrophages *in vitro* (Fig. 2). Interestingly, Galf exposure enhanced the expression of a panel of immune genes in infected macrophages, whereas no effect was recorded in uninfected cells. A major induction of genes encoding M1 pro-inflammatory cytokines was observed in Galf-treated cells, such as IL-12 (Mann-Whitney test: *U* = 150.0, *P* = 0.0010), IL-1β (*U* = 75.0, *P* < 0.0001) and TNF-α (*U* = 174.5, *P* = 0.0076), as well as iNOS, involved in the oxidative burst (*U* = 52.0, *P* = 0.0044). Similarly, monocyte and lymphocyte chemokines MCP-1 and CXCL-10 genes were strongly overexpressed compared to the control condition. Besides, macrophages also revealed a strong induction of the M2 cytokine IL-10, in response to Galf treatment.

### Galf-treated mice display an enhanced Th1 immune response (in target organs but reduced serum inflammatory cytokines)

**Fig. 3:**
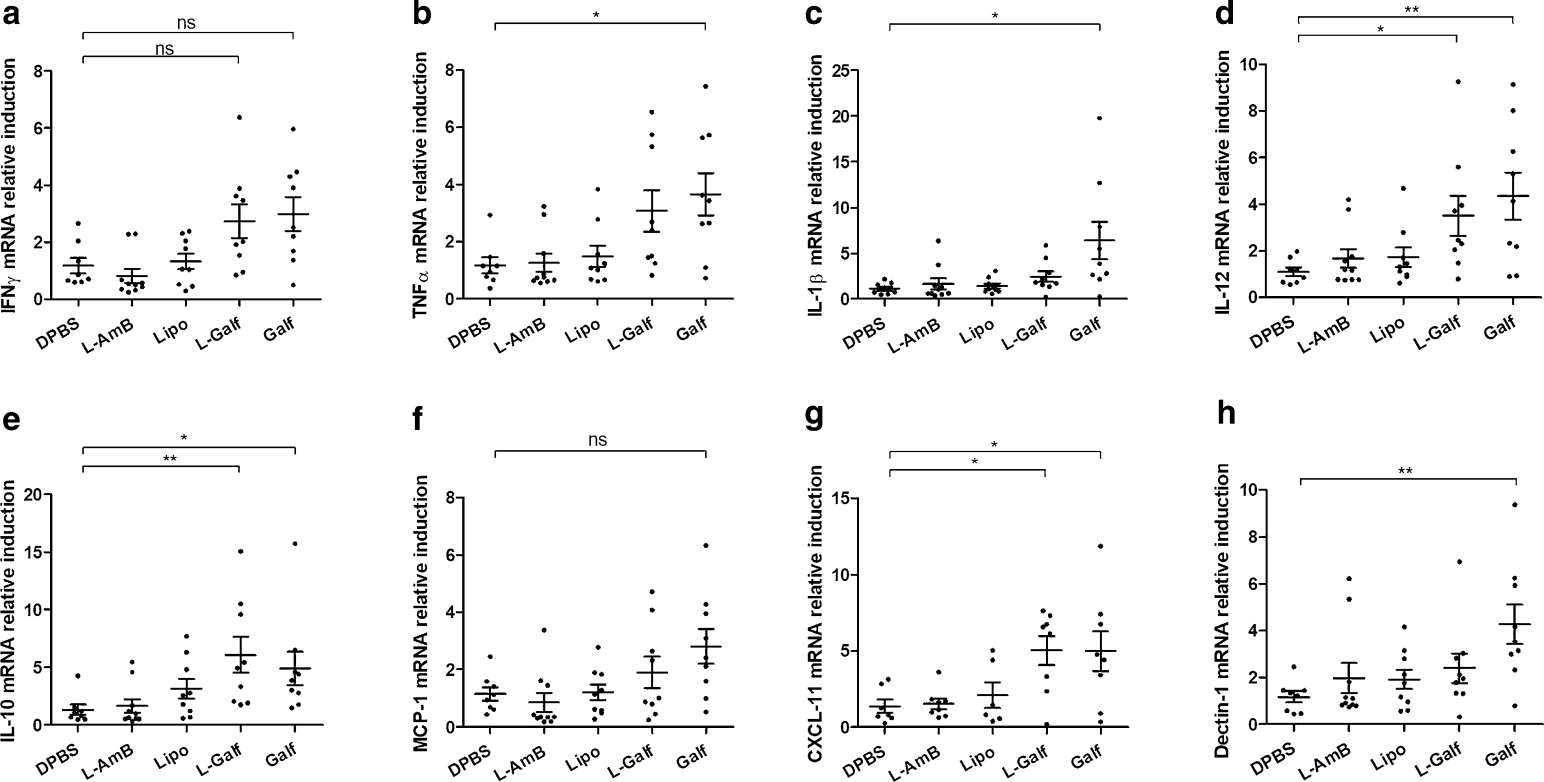
Effect of Galf formulations on mRNA expression of splenic immune markers in *L. donovani*-infected mice. mRNA expression of IFN-γ (**a**), TNF-α (**b**), IL-1β (**c**), IL-12 (**d**), IL-10 (**e**), MCP-1 (**f**), CXCL-11 (**g**) and Dectin-1 (**h**) was evaluated. BALB/c mice were infected intraperitoneally with *L. donovani* promastigotes and treated from day 14 to day 21 with DPBS (control group), liposomal Amphothericin B (L-AmB), free Galf, liposomal Galf (L-Galf) or empty liposomes (Lipo). Animals were sacrificed 7 days after the end of treatment (day 28). Total RNA was extracted and purified from splenic tissue and quantified by comparison to 18S RNA. Each group contained 9 or 10 mice. Mean inductions* ± *SE are represented by cross bars. Results from treated mice were compared to untreated mice using the non-parametric Kruskal–Wallis test (*P ≤ 0.05, **P ≤ 0.01)

mRNA induction of main immune effectors was quantified in the spleen, the liver and the BM and showed contrasting results according to treatment. Treatment with L-AmB or empty liposomes did not impact the expression of splenic immune markers (Fig. 3). By contrast, the expression of TNF-α, IL-1β and IL-12 mRNA was significantly induced in the spleen of Galf-treated mice, compared to control mice, as shown by the mean respective induction rates of 3.66 ± 0.74 *vs* 1.18 ± 0.27, 6.40 ± 2.08 *vs* 1.15 ± 0.22 and 4.35 ± 1.00 *vs* 1.10 ± 0.19, respectively (Fig. 3b–d). L-Galf treatment also significantly induced IL-12 mRNA expression. Although not statistically significant, the induction of IFN-γ increased by a factor of 2.3 and 2.5, respectively, in L-Galf and Galf-treated groups. An induction of the chemokine CXCL-11 by Galf and L-Galf, was also observed (Kruskal–Wallis test: *χ*^2^ = 10.23, *df* = 4, *P* = 0.0397) (Fig. 3g).

Similar to previous results obtained on human macrophages, a strong increase in the expression of the immunoregulatory cytokine IL-10 was observed in both Galf and L-Galf groups, compared to control group (Kruskal–Wallis test: *χ*^2^ = 17.92, *df* = 4, *P* = 0.0223 and *P* = 0.0057, respectively) (Fig. 3e). Finally, Galf treatment also triggered an enhanced expression of the macrophage receptor Dectin 1 gene (Kruskal–Wallis test: *χ*^2^ = 11.73, *df* = 4, *P* = 0.0078) (Fig. 3h).

**Fig. 4:**
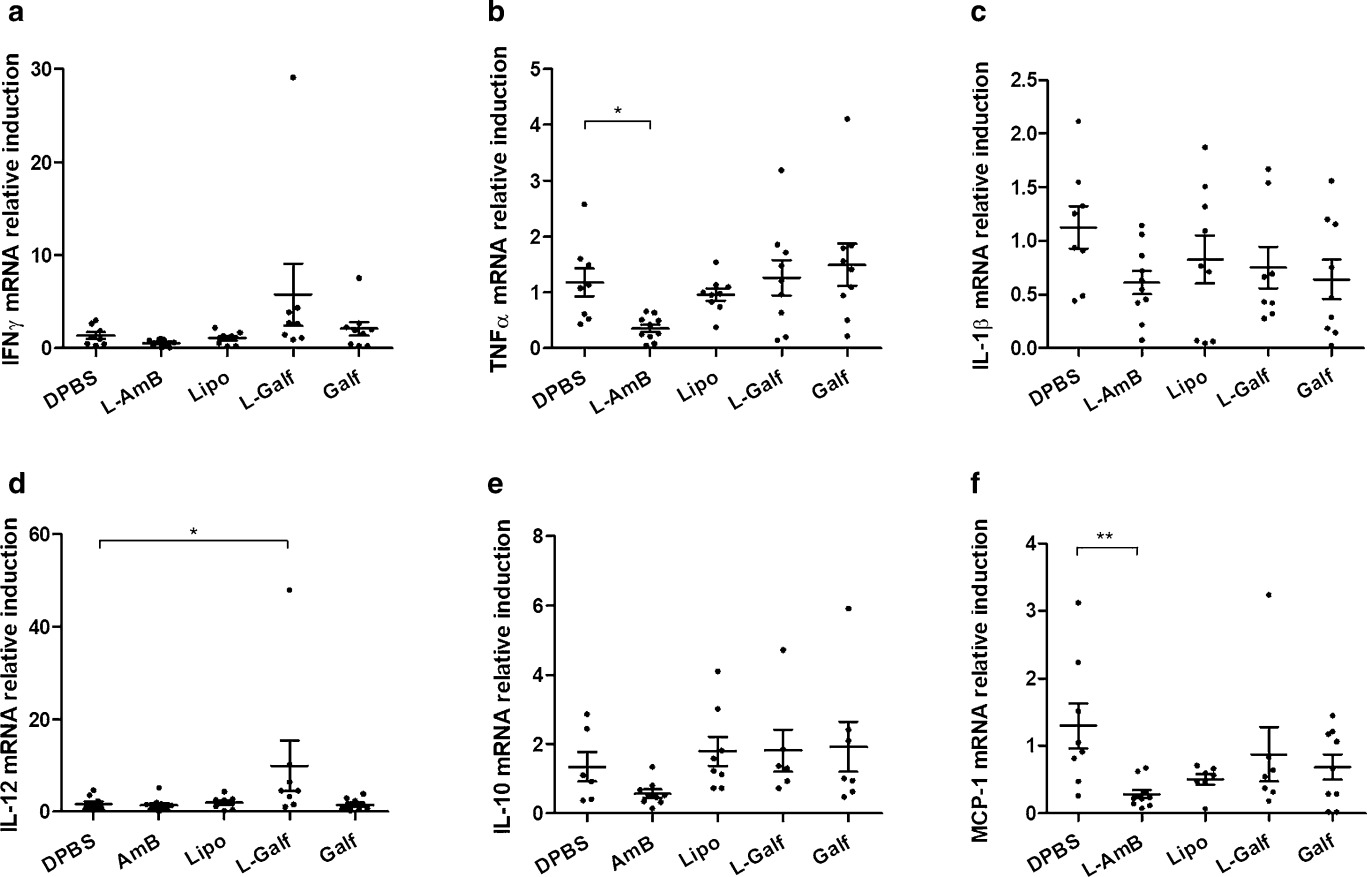
Effect of Galf formulations on mRNA expression of hepatic immune markers in *L. donovani*-infected mice. mRNA expression of IFN-γ (**a**), TNF-α (**b**), IL-1β (**c**), IL-12 (**d**), IL-10 (**e**) and MCP-1 (**f**) was evaluated. BALB/c mice were infected intraperitoneally with *L. donovani* promastigotes and treated from day 14 to day 21 with DPBS (control group), liposomal Amphothericin B (L-AmB), free Galf, liposomal Galf (L-Galf) or empty liposomes (Lipo). Animals were sacrificed 7 days after the end of treatment (day 28). Total RNA was extracted and purified from liver tissue and quantified by comparison to 18S RNA. Each group contained 9 or 10 mice. Mean inductions* ± *SE are represented by cross bars. Results from treated mice were compared to untreated mice using the non-parametric Kruskal–Wallis test (*P ≤ 0.05, **P ≤ 0.01)

In the liver, only a discrete immunomodulatory effect was noticed. An induction of IL-12 expression was observed after L-Galf treatment (9.93 ± 5.52 *vs* 1.63 ± 0.59, Kruskal–Wallis test: *χ*^2^ = 10.85, *df* = 4, *P* = 0.0457) (Fig. 4d); the same trend was observed for IFN-γ (Fig. 4a). Again, empty liposomes did not modify induction of immune effectors. Conversely, L-Amb significantly repressed Th1 inflammatory markers, such as MCP-1 (Kruskal–Wallis test: *χ*^2^ = 10.89, *df* = 4, *P* = 0.046) and TNF-α (Kruskal–Wallis test: *χ*^2^ = 14.27, *df* = 4, *P* = 0.0200) (Fig. 4).

**Fig. 5:**
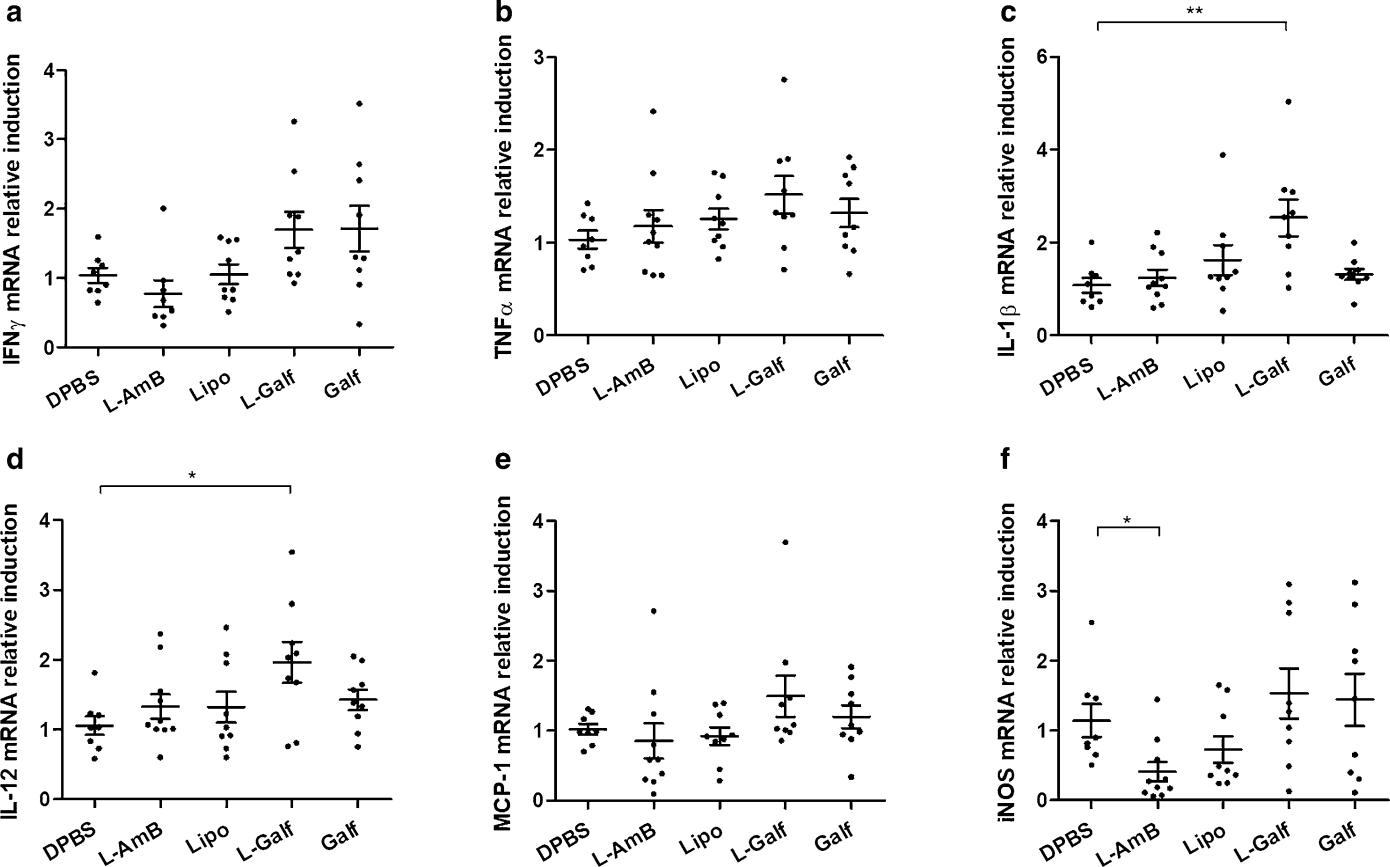
Effect of Galf on mRNA expression of immune markers in bone marrow cells from *L. donovani*-infected mice. mRNA expression of IFN-γ (**a**), TNF-α (**b**), IL-1β (**c**), IL-12 (**d**), MCP-1 (**e**) and iNOS (**f**) was evaluated. BALB/c mice were infected intraperitoneally with *L. donovani* promastigotes and treated from day 14 to day 21 with DPBS (control group), liposomal Amphotericin B (L-AmB), free Galf, liposomal Galf (L-Galf) or empty liposomes (Lipo). Animals were sacrificed 7 days after the end of treatment (day 28). Total RNA was extracted and purified from bone marrow cells and quantified by comparison to 18S RNA. Each group contained 9 or 10 mice. Mean inductions ± SE are represented by cross bars. Results from treated mice were compared to untreated mice using the non-parametric Kruskal–Wallis test (*P ≤ 0.05, **P ≤ 0.01)

Cytokine expression in BM cells displayed a similar trend after L-Galf treatment, with a statistically significant increase of IL-1β (Kruskal–Wallis test: *χ*^2^ = 12.28, *df* = 4, *P* = 0.0080) and IL-12 (Kruskal–Wallis test: *χ*^2^ = 6.574, *df* = 4, *P* = 0.0300), as well as a moderate increase of IFN-γ, TNF-α and MCP-1 (ns) (Fig. 5).

**Fig. 6:**
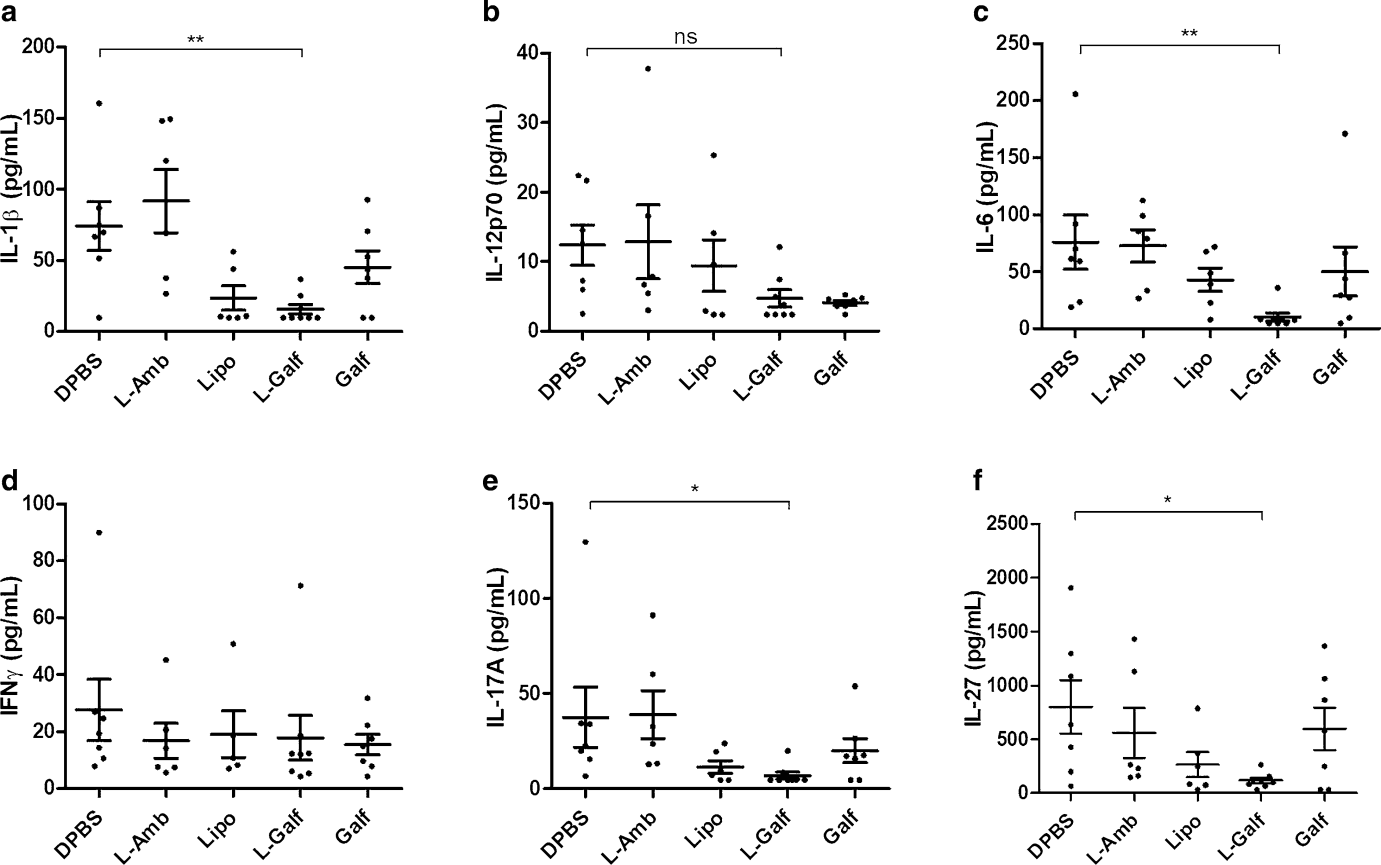
Serum cytokines levels after Galf treatment in *L. donovani* infected mice. Quantification of serum IL-1 β (**a**), IL-12p70 (**b**), IL-6 (**c**), IFN-γ (**d**), TNF-α (**e**), IL-17A (**f**), IL-27 (**g**) and MCP-1 (**h**) at the end of treatment (day 21). BALB/c mice were infected with *L. donovani* promastigotes, and treated from day 14 to day 21 with indicated treatments. Blood samples were collected at the end of treatment (day 21). Titrations were performed in duplicate using a bead-based immunoassay Legendplex® multi-analyte flow assay kit (BioLegend). Mean cytokine concentration ± SE (pg/ml) are represented by cross bars. Data are representative of one experiment. Each group contained 9 or 10 mice. Results from treated mice were compared to untreated mice using the non-parametric Kruskal–Wallis test (*P ≤ 0.05, **P ≤ 0.01)

Interestingly, serum levels of inflammatory cytokines (IL-1β, IL-6, IL-27, and IL-17A) significantly decreased at the end of treatment by L-Galf, but Th1 cytokines IFN-γ and IL-12 remained unchanged (Fig. 6).

### Moderate hepatic and splenic histological modifications

**Fig. 7:**
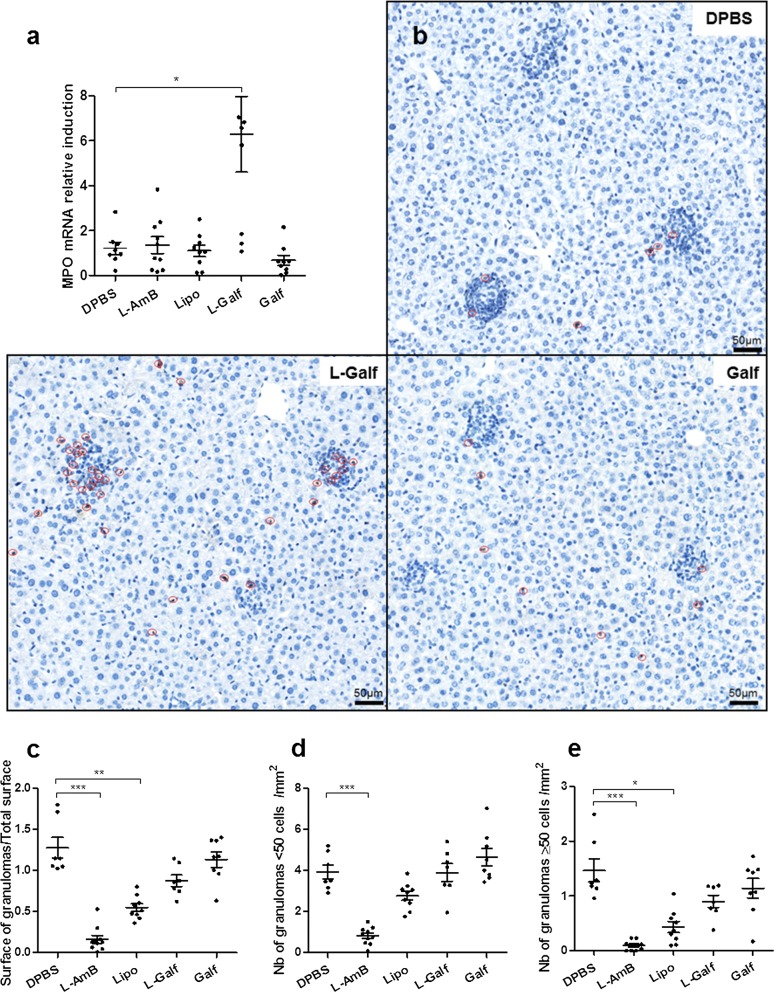
Myeloid cell attraction by Galf formulations and quantification of the granulomatous response in the liver. **a** Relative expression of MPO mRNA in liver according to treatment. **b** Immunohistochemical staining of MPO-positive cells in the liver. Representative microscopic fields according to treatment group. **c** Ratio of the granulomas surface by the total surface of liver section. **d** Number of small granulomas (< 50 cells) per mm^2^ of liver tissue. **e** Number of large granulomas (≥ 50 cells) per mm^2^ of liver tissue. Slide images were obtained using the NanoZoomer (Hamamatsu Photonics), and analyzed using the NDP.view2 viewing software (Hamamatsu Photonics) for granuloma measurement, quantification and size analysis. Each group contained 9 or 10 mice. Results from treated mice were compared to untreated mice using the non-parametric Kruskal–Wallis test (*P ≤ 0.05, **P ≤ 0.01, ***P ≤ 0.001)

Interestingly, a marked increase of myeloperoxidase (MPO) mRNA induction was observed in both the liver (Fig. 7a) and the spleen (not shown) of L-Galf-treated mice, compared to control group (6.29 ± 1.68 *vs* 1.22 ± 0.27 and 4.48 ± 1.08 *vs* 1.04 ± 0.13, respectively, Kruskal–Wallis test: *χ*^2^ = 16.55, *df* = 4, *P* = 0.0310 and *χ*^2^ = 14.89, *df* = 4, *P* = 0.0043, respectively). To analyze further whether this MPO induction was due to increased oxidative burst effectors or to the recruitment of myeloid cells, immunohistochemical staining was performed on liver sections to reveal myeloid cells, using an anti-MPO antibody. Microscopical examination revealed a higher number of MPO-positive cells in the liver of mice treated with L-Galf, compared to controls (Fig. 7b).

The examination of the hepatic tissue response after H&E staining revealed no impact of treatment on the liver surface covered by granulomas (Fig. 7c), neither on the number of hepatic granulomas, whatever the granuloma size (Fig. 7d, e). By contrast, the proportion of liver area covered by granuloma structures and the number of large granulomas (≥ 50 cells) was considerably reduced in AmB treated group (Kruskal–Wallis test: *χ*^2^ = 33.14, *df* = 4, *P* < 0.0001 and *χ*^2^ = 28.81, *df* = 4, *P* < 0.0001, respectively), and to a lesser degree in Lipo-treated mice (Kruskal–Wallis test: *χ*^2^ = 33.14, *df* = 4, *P* = 0.0090 and *χ*^2^ = 28.81, *df* = 4, *P* = 0.0097, respectively).

**Fig. 8:**
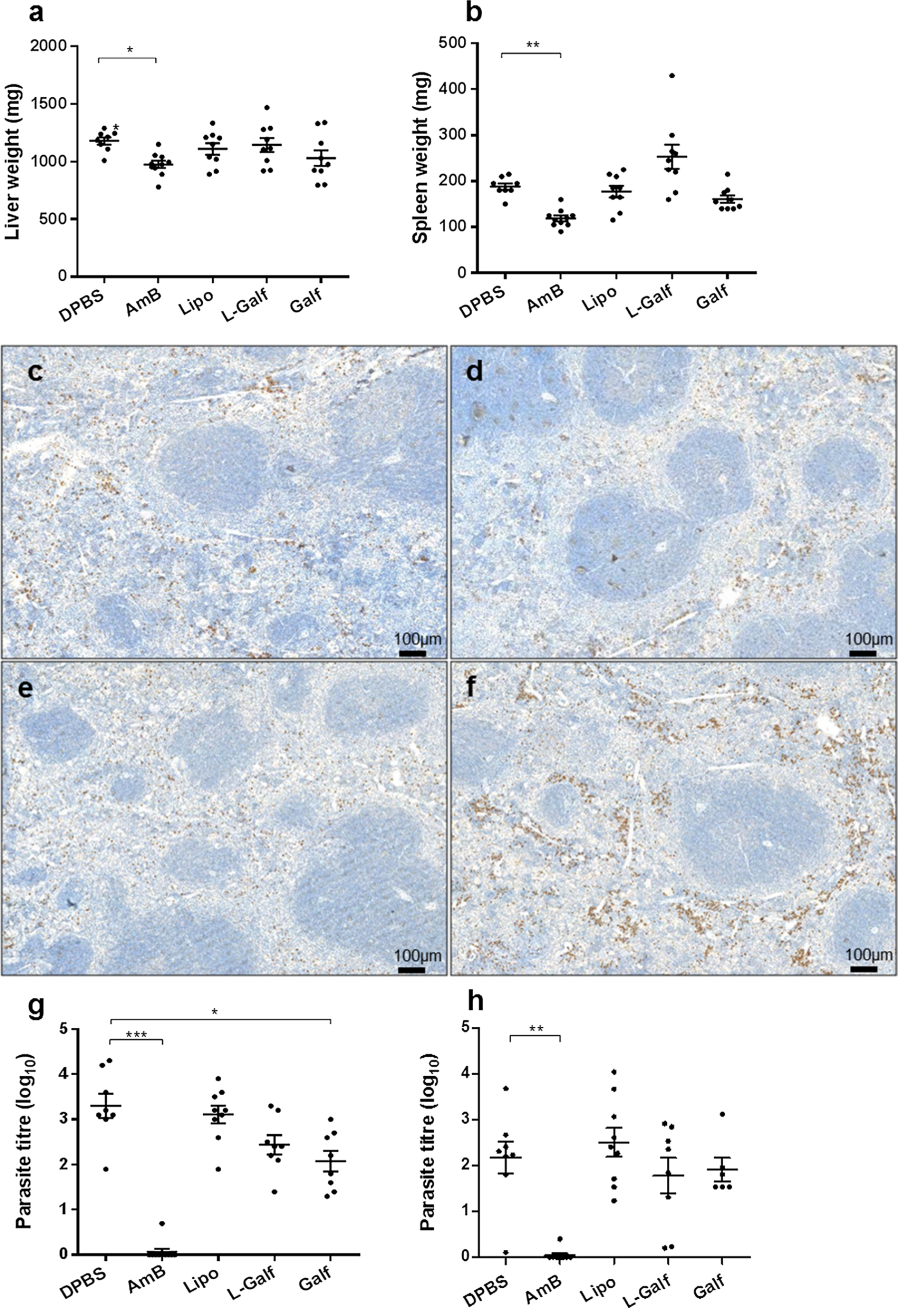
Organ weight and parasite burden according to treatment. Liver (**a**) and spleen (**b**) weight at sacrifice. CD8^+^ cell staining in the spleen from control mice (**c**), Lipo- (**d**), Galf- (**e**) and L-Galf- (**f**) treated mice. Parasite burdens in liver (**g**) and spleen (**h**). *Leishmania donovani*-infected mice were infected intraperitoneally with *L. donovani* promastigotes, treated from day 14 to day 21 with indicated treatments, and sacrificed 7 days after the end of treatment (day 28). After homogenization of standardized biopsies of liver and spleen, parasite loads were evaluated using a dilution assay. Results were expressed as the log of the titer, adjusted per milligram of organ. Means ± SE are represented by cross bars. Data are representative of one experiment. Each group contained 9 or 10 mice. Results from treated mice were compared to untreated mice using the non-parametric Kruskal–Wallis test (*P ≤ 0.05, **P ≤ 0.01, ***P ≤ 0.001)

Although not statistically significant, a splenomegaly was noticed in L-Galf-treated mice (mean weight 253 ± 26 mg *vs* 188 ± 7 mg for control mice), whereas liver weights remained unchanged (Fig. 8a, b). This organomegaly could not be linked clinically, to a higher clinical disease progression, as all animals remained healthy during the timespan of the experiment, with no loss of body weight. To confirm whether splenomegaly could be due to cell attraction in relation with induced Th1 response, we performed tissue staining to estimate CD4+ T cells, CD8+ T cells, and CD20+ B lymphocyte infiltrates. Interestingly, only a marked increase of CD8+ T cells was observed in L-Galf treated mice (Fig. 8c–f).

Of note, biochemical serum dosages did not reveal any renal or hepatic toxicity (Additional file 2: Figure S1). Serum creatinine levels remained unchanged by treatments. Mice treated with liposomal formulations displayed a mild increase in alanine transaminase (ALT) levels, reaching 45.8 ±2 .6 U/l (Kruskal–Wallis test: *χ*^2^ = 14.73, *df* = 4, *P* = 0.043) and 54.9 ± 5.5 U/l (Kruskal–Wallis test: *χ*^2^ = 14.73, *df* = 4, *P* = 0.0010) with L-Amb and L-Galf, respectively, compared to DPBS-treated mice (34.2 ± 3.0 U/l). By contrast, alkaline phosphatase (ALP) levels decreased in the L-Galf group (120.5 ± 7.3 *vs* 176.4 ± 8.8 U/l, Kruskal–Wallis test: *χ*^2^ = 16.54, *df* = 4, *P* = 0.0017).

### Galf and L-Galf treatments have moderate impact on parasite loads *in vivo*

Parasite burden was determined in liver and spleen by microtitration (Fig. 8g, h). In the liver, Galf significantly decreased the parasite loads (mean log titers of 2.07 ± 0.23 *vs* 3.30 ± 0.27 in the control group, Kruskal–Wallis test: *χ*^2^ = 30.21, *df* = 4, *P* = 0.0430), while only a moderate anti-parasitic effect was observed in the spleen. By contrast, the parasite was drastically cleared from the liver and the spleen of L-Amb-treated mice (mean log titers of 0.07 ± 0.07 and 0.04 ± 0.04, respectively), which was correlated to reduced organ weights (Kruskal–Wallis test: *χ*^2^ = 9.262, *df* = 4, *P* = 0.0261 and *χ*^2^ = 27.81, *df* = 4, *P* = 0.0022, respectively, Fig. 8a, b). Both Galf treatments had nearly no effect on parasite loads in the bone marrow (data not shown).

## Discussion

Interest for immunomodulatory drugs is currently increasing, as possible anti-*Leishmania* adjuvant treatments. Whereas immunostimulation could be a promising strategy to enhance efficacy and/or reduce drug intakes, it should be adapted to the clinical setting, as the expected benefit may vary according to the cutaneous or visceral setting of the disease. In PKDL or cutaneous leishmaniasis, excessive inflammatory response may worsen the prognosis, whereas in VL, a restoration of Th1 response is necessary to cure the patient [34]. It is now well recognized that the M1/M2 macrophage polarization state is tightly dependent on the tissue microenvironment and influences its microbicidal activity [35, 36]. Indeed, a feature of macrophages is their dynamic plasticity, expressed by their ability to alter their phenotype, depending on the cytokine microenvironment, and to polarize towards distinct polarization states. In response to IFN-γ or TNF-α and to the recognition of danger signals, M1-activated macrophages develop a high phagocytic potential, enhancing the clearance of intracellular pathogens, while skewing a polarization toward a M2 subset may benefit infections, limiting pro-inflammatory processes. Macrophages polarization has also been shown to be tightly dependent on metabolic programs involving various carbohydrates, raising the therapeutic potential of such components to modulate macrophage responses [37].

Here, the observation that Galf could trigger mRNA induction of pro-inflammatory cytokines (IL-1β and IL-12) in macrophage cells *in vitro* was indicative of activation towards M1 phenotype. The significant decrease in parasite proliferation obtained in human macrophages treated with Galf, and the enhanced level of NF-κB mRNA induction in macrophages was consistent with M1 polarization. Indeed, the activation of the NF-κB signaling pathway is known to be a prerequisite to initiate innate immune responses and protective Th1-mediated immunity [38]. Among others, this primordial mechanism was shown to be impaired during *Leishmania* infection, leading to macrophage tolerance and hypo-responsiveness that could be reversed by using such immunostimulating furanosides. These observations are consistent with recent evidence indicating that the state of macrophage polarization plays a critical role in the control of infections [22, 39–45] and the regulation of inflammatory processes [46]. Recently, El Hajj et al. [47] showed that imiquimod and an imiquimod analog (EAPB0503) had an antiparasitic effect associated with a strong stimulation of the NFκB pathway, leading to a dramatic increase of Th1 cytokines and a decrease of Th2 cytokines. These observations led us to investigate the immunomodulatory properties of Galf in a mouse model treated with the reference drug for VL treatment, L-AmB. We used a duration and route of treatment similar to that used for L-AmB, in the aim to assess whether an effect could be obtained with a short treatment course.

*In vivo* we observed a similar response, as previously obtained *in vitro*, showing enhancement of a Th1 response in all target organs, including induction of key pro-inflammatory cytokines and associated effectors (iNOS). In the liver, MPO-positive cells were recruited in granulomas, an event previously identified as crucial for early parasite control [48]. Here, while L-Galf displayed a moderate Th1 immune response in the liver, both treatments decreased parasite replication (1 log_10_), although only statistically significantly with Galf (Fig. 8). The anti-parasitic response was not correlated with the number of granulomas, as already described [49], particularly for L-AmB. This could be explained firstly by the fact that the early anti-parasitic effect decreases the number of infected foci and therefore the number of granulomas formed around infected Küpffer cells. Secondly, it could also be due to the use of liposomes, as empty liposomes reduced the formation of granulomas. A recent study reported that the use of liposomal formulations of meglumine antimoniate was associated with a reduced hepatic tissue response and increased Th1 response [50], which is consistent with the slightly lower granulomatous response observed in mice treated with L-Galf compared to Galf in the present study.

In contrast to the liver, the immunostimulatory effect of both Galf treatments was significant in the spleen, but the anti-parasitic effect was only mild. The splenomegaly observed in L-Galf-treated mice could be related to T lymphocyte recruitment promoted by the enhanced expression of CXCL-11, and is supported by the influx of CD8 cells observed on tissue sections. This chemokine is redundant with CXCL-9 and CXCL-10 and can also interact with CXCR3-positive cells to promote Th1 cell maturation, which has been shown to contribute to parasite control in BALB/c mice [51, 52]. A recent study using CXCL-9- or CXCL-10-deficient mice has shown the contribution of these chemokines to parasite control in the liver [49].

Interestingly, Galf exposure triggered the induction of Dectin-1 gene expression in the spleen, which was also found in peritoneal macrophages (data not shown). Among other C-type lectin receptors characterizing a M2b polarization such as Mannose receptor, Dectin-1 is a β-glucan macrophage receptor that currently raises much interest in understanding mechanisms involved in early innate immunity and activation of adaptive defense in response to various microorganisms, including mycobacteria [53], fungi [54] and *Leishmania*. Indeed, in a *L. infantum*-infected murine model, Dectin-1 was shown to be crucial for the initial *Leishmania* recognition by macrophages, and subsequent activation of the defense pathways, toward the activation of Syk (spleen tyrosine kinase)-p47 phox axis needed for ROS generation and resistance to infection [22]. Other experiments have supported this finding and further evidenced the critical role of Dectin-1 for inflammasome activation, restriction of parasite replication in macrophages and mouse resistance to *L. amazonensis* infection [55].

The concomitant induction of the IL-10 gene observed both in infected human macrophages and splenic tissues of Galf-treated mice could be considered, at first glance, contradictory with the aforesaid results. Such increased induction of both IFN-γ and IL-10 has been described in other therapeutic evaluations as with ursolic acid in a VL model of *L. infantum*-infected hamsters [56]. The immunosuppressive function of IL-10 has been fully evidenced in the past in experimental and human VL, showing a strong correlation between IL-10 levels and disease severity and outcome [57–59]. This involves in particular the deactivation of the leishmanicidal macrophage functions, the down- regulation of antigen presentation mechanisms and the decrease of IFN-γ production in T cells [60]. However, this last point also emphasizes the crucial role of IL-10 to counteract an excessive inflammatory response, which would be pernicious for the host. As illustrated in cutaneous leishmaniasis (CL), plasmatic IL-10 levels are lower in patients with severe mucosal leishmaniasis (ML) compared to those with CL, suggesting the protective role of IL-10 [61]. Downregulation of IL-10 reported in ML was associated with a poorer ability to prevent exaggerated inflammatory response and tissue damages [61, 62]. Besides, an increase in the level IL-10 in association with a reduction in the IFN-γ/IL-10 ratio is commonly described in cured CL compared to active CL. IL-10 production has also been negatively correlated to disease duration in a cohort of ML patients [63].

Here we could suspect the pro-inflammatory response enhanced by Galf exposure to trigger an IL-10 mediated immunosuppressive compensator mechanism to control the inflammation. IL-10 can be produced by several cell types including macrophages, B cells and regulatory CD4+ T cells (T reg). Among T reg, the antigen-driven CD25^-^/Foxp3^-^ T cell subset can produce large amounts of IL-10 in the spleen during VL [64]. Here, Foxp3 gene expression remained unchanged in the spleen of Galf-treated mice (data not shown), ruling out T reg involvement. Thus we could suspect that Galf exposure more likely potentiates the activation of splenic T cell subsets secreting both IFN-γ and IL-10, as already described in several mouse models infected with *L. major* and other pathogens [58].

Moreover, L-Galf-treated mice displayed strongly lowered blood pro-inflammatory cytokines levels, contrasting with hepatic and splenic response. Such a decrease is consistent with data from previous *L. infantum*-infected human cohorts, showing a significant drop of circulating cytokines such as IFN-γ, IL-6, TNF-α and IL-10 after treatment, correlated with healing [65]. Interestingly, a similar decrease in IL-27 level was observed, a cytokine previously known to be involved in susceptibility to VL, in humans and murine models [65–68]. Produced by myeloid cells (mainly dendritic cells and macrophages), IL-27 plays a complex immunoregulatory role, exerting both pro- and anti-inflammatory effects in various diseases [69]. While IL-27 is crucial to prevent severe tissue damage related to inflammation during the initial stages of VL [67], it also favors parasite persistence, as evidenced by the higher resistance of IL-27R-deficient mice, or after IL-27 neutralization [66–68].

By comparison to *in vitro* results, Galf treatment showed only limited effects on parasite growth *in vivo* (one log_10_ reduction). A PK/PD issue cannot be ruled out, and studies on furanoside diffusion in tissues and metabolism are needed. Determination of Galf concentrations in target tissues would provide useful data to exclude an infra-therapeutic dosing. Nonetheless, higher dosing could be easily considered given the high safety of this drug. Previous evaluation on *in vitro* human macrophages revealed an excellent selectivity index, as high as 160 [28], and in mice, serum biochemical dosages ruled out kidney or hepatic toxicity.

Interestingly, no immunomodulatory effect was induced by L-AmB treatment which is consistent with previous reports showing the low immunomodulatory potential of this drug [58]. Hence, our data indicate that the combination of Galf with a conventional anti-*Leishmania* drug should be investigated as an enhancer of immune restoration during VL, using an immunosuppressed murine model.

## Conclusions

Taken together, this study brings new insights into the manipulation of the host immune system to fight visceral leishmaniasis, using small synthetic furanosides. In the era of new biotherapies and personalized medicine, such a boost of the immune system combined with an anti-parasitic molecule could help counterbalance the immune downregulation responsible for clinical relapses of VL in immunocompromised patients, a clinical situation which is frequent in high-income countries, but also in Africa.

## Supplementary information

**Additional file 1: Table S1.** Sequences of the oligonucleotides used for real-time PCR.

**Additional file 2: Figure S1.** Levels of serum creatinine, ALT and ALP after treatment. Mouse blood was collected at the end of treatment (day 21) to monitor renal toxicity using creatinine dosage (**a**), and liver damages by measuring transaminases (ALT) (**b**) and alkaline phosphatase (ALP) (**c**) levels. Each group contained 9 or 10 mice. Mean serum concentration or titer ± SEM are represented by cross bars. Data are representative of one experiment. Results from treated mice were compared to untreated mice using the non-parametric Kruskal–Wallis test. *P ≤ 0.05, **P ≤ 0.01.

## Data Availability

Data supporting the conclusions are included within the article and its additional files. Detailed data are available from the corresponding author upon request.

## Abbreviations

ALP: alkaline phosphatase
ALT: alanine transaminase
BM: bone marrow
CL: cutaneous leishmaniasis
CXCL: (C-X-C motif) ligand
DC-SIGN: dendritic cell- specific intercellular adhesion molecule-3 grabbing non- integrin
FCS: fetal calf serum
Galf: octyl-β-D-galactofuranose
GIPLs: glycosylinositol phospholipids
Gluc: meglumine antimoniate
H&E: hematoxylin and eosin
HePC: miltefosine
HM: human monocyte-derived macrophages
IFN-γ: interferon γ
IL: interleukin
iNOS: inducible nitric oxide synthase
L-AmB: liposomal amphothericin B
L-Galf: liposomal octyl-β-d-galactofuranose
Lipo: empty liposome
LPG: lipophosphoglycan
MCP-1: monocyte chemoattractant protein 1
MGG: May–Grünwald–Giemsa
ML: mucosal leishmaniasis
MPO: myeloperoxidase
PKDL: post-kala-azar dermal leishmaniasis
ROS: reactive oxygen species
SE: standard error
SIGN-R1: specific intercellular molecule-3-grabbing nonintegrin-related 1
T reg: regulatory T cell
TNF-α: tumor necrosis factor α
VL: visceral leishmaniasis
