# Supplementary material for: In vitro and in vivo immunomodulatory properties of octyl-β-d-galactofuranoside during Leishmania donovani infection
Source: Parasit Vectors. 2019 Dec 23;12:600. doi: 10.1186/s13071-019-3858-0 (PMC6929453; doi:10.1186/s13071-019-3858-0)
Supplement: Supplementary file 1 — Additional file 1: Table S1. Sequences of the oligonucleotides used for real-time PCR. [file 13071_2019_3858_MOESM1_ESM.docx]

**Additional file 1: Table S1****.** Sequences of the oligonucleotides used for real-time PCR.

| **Targeted gene** | | **Forward primer (5’-3’)** | **Reverse primer (5’-3’)** |
| --- | --- | --- | --- |
| Human |  |  |  |
|  | 18S rRNA | GTAACCCGTTGAACCCCATT | CCATCCAATCGGTAGTAGCG |
|  | IL-12 | TCAGTTTGGCCAGAAACCRC | GTTTGTCTGGCCTTCTGGAG |
|  | TNF-α | AACCTCCTCTCTGCCATCAA | ATGTTCGTCCTCCTCACAGG |
|  | IL-1β | TCATGCTCTGTTCTTGGGAAT | GCTTGTCCTGCTTTCTGTTC |
|  | IL-10 | ATTGGTGAAACCCCGTCTCTAC | TTCCATCTCCTGGGTTCAAG |
|  | MCP-1 | AGTCTCTGCCGCCCTTCT | GTGACTGGGGCATTGATTG |
|  | CXCL-10 | CCACGTGTTGAGATCATTGGC | TTCTTGATGGCCTTCGATTC |
|  | NFκBp65 | TCTGCTTCCAGGTGACAGTG | ATCTTGAGCTCGGCAGTGTT |
|  | iNOS | ACTGGAGCTAAAGTGGTACG | ATGTTGATCTCAACGACAGC |
| Mouse |  |  |  |
|  | 18S rRNA | TTGGCAAATGCTTTCGCTC | CGCCGCTAGAGGTGAAATTC |
|  | IL-12p35 | CTCCTGTGGGAGAAGCAGAC | GATAGCCCATCACCCTGTTG |
|  | IFNγ | CCCTATGGATGACGGAGA | CTGTCTGCTGGTGGAGTTCA |
|  | TNF-α | TAGCTCCCAGAAAAGCAAGC | TTT TCT GGA GGG AGA TGT GG |
|  | IL-1β | GATCCACACTCTCCAGCTGCA | CAACCAACAAGTGATATTCTCCA |
|  | IL-10 | CTCATGGGTCTTGGGAAGAG | AACTGGCCACAGTTTTCAGG |
|  | MCP-1 | TCTGGACCCATTCCTTCTTG | AGGTCCCTGTCATGCTTCTG |
|  | CXCL-11 | GCTGAGTGCTTTCACCTTCC | GGAATTCCCCTCCTTGACTC |
|  | iNOS | GTGGTGACAAGCACATTTGG | AAGGCCAAACACAGCATACC |
|  | Dectin1 | CATCGTCTCACCGTATTAATGCAT | CCCAGAACCATGGCCCTT |
|  | MPO | AAGGCCTGTCTCTGCTGTTTAC | ATTGGCACTCAGTTTAGGAAGC |
