# Supplementary figures and images for: In vitro and in vivo immunomodulatory properties of octyl-β-d-galactofuranoside during Leishmania donovani infection
Source: Parasit Vectors. 2019 Dec 23;12:600. doi: 10.1186/s13071-019-3858-0 (PMC6929453; doi:10.1186/s13071-019-3858-0)

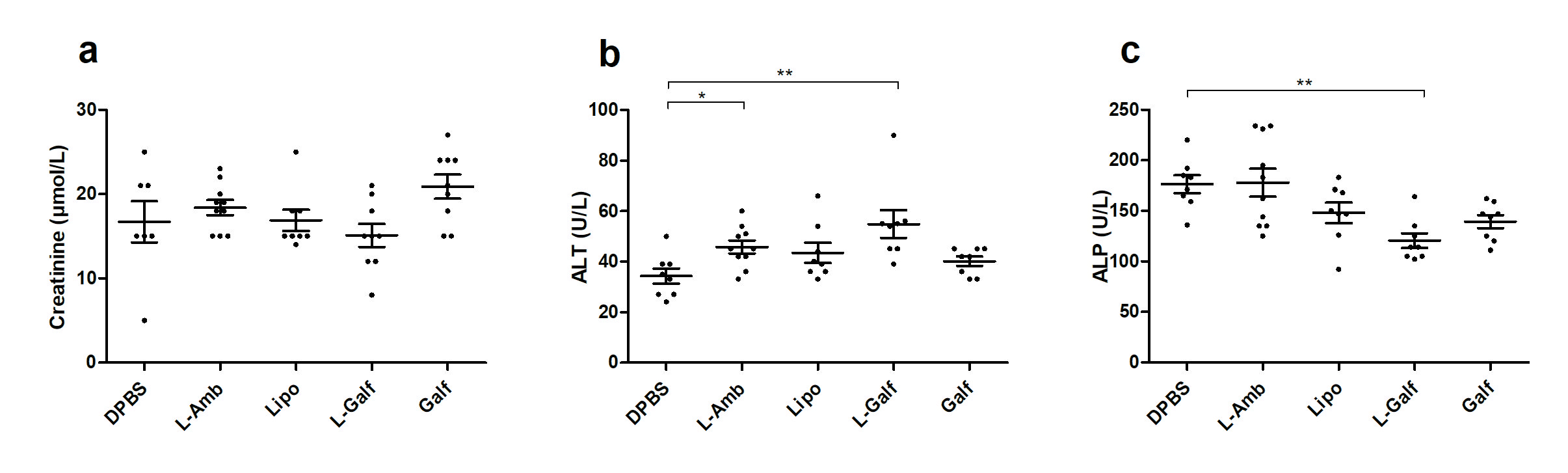

Supplement: Supplementary file 2 — Additional file 2: Figure S1. Levels of serum creatinine, ALT and ALP after treatment. Mouse blood was collected at the end of treatment (day 21) to monitor renal toxicity using creatinine dosage (a), and liver damages by measuring transaminases (ALT) (b) and alkaline phosphatase (ALP) (c) levels. Each group contained 9 or 10 mice. Mean serum concentration or titer ± SEM are represented by cross bars. Data are representative of one experiment. Results from treated mice were compared to untreated mice using the non-parametric Kruskal–Wallis test. *P ≤ 0.05, **P ≤ 0.01. [file 13071_2019_3858_MOESM2_ESM.tif]
